# Size-Selective Nanoporous Atomically Thin Graphene Separators for Lithium–Sulfur Batteries

**DOI:** 10.1021/acsami.5c11148

**Published:** 2025-09-04

**Authors:** Daniel A. Gribble, Peifu Cheng, Vilas G. Pol, Piran R. Kidambi

**Affiliations:** † Davidson School of Chemical Engineering, 8522 Purdue University, West Lafayette, Indiana 47907, United States; ‡ Department of Chemical and Biomolecular Engineering, 5718 Vanderbilt University, Nashville, Tennessee 37212, United States; ∇ Department of Mechanical and Aerospace Engineering, University of Florida, Gainesville, Florida 32611, United States

**Keywords:** nanoporous atomically thin membranes (NATMs), graphene, graphene membranes, modified separator, battery separator, lithium−sulfur batteries, lithium polysulfide

## Abstract

Lithium–sulfur batteries (LSBs) are extensively researched for their high energy densities but are hindered by the lithium polysulfide (LiPS) shuttling effect, which results in poor cyclability. A popular mitigation strategy is separator modification, where a LiPS trapping material is slurry-coated onto a conventional microporous polypropylene (PP) separator. This additional mass and volume unfortunately compromise the overall energy density of the LSB. This study aims to take a separator modification approach that avoids this issue. Nanoporous atomically thin membranes (NATMs) made of graphene are gaining attention for their scalable synthesis, tunable pore size, and negligible pore length. Herein, we apply a well-characterized graphene NATM for reasons similar to those of a size-selective interlayer in LSBs. The tailored pore size of ∼0.7–1.0 nm and atomic thinness facilitate the passage of Li^+^ (solvated ionic diameters ∼0.54–1.26 nm) and blockage of larger LiPS (solvated ionic diameters ∼0.81–1.69 nm) without adding significant impedances or mass. The sulfur confinement is confirmed through scanning electron microscopy and energy-dispersive X-ray spectroscopy elemental analysis of the Li anode. An LSB with a NATM@PP separator shows virtually no capacity loss over 150 cycles, demonstrating efficacy of size-selective molecular sieving using NATMs in LSBs.

## Graphical abstract

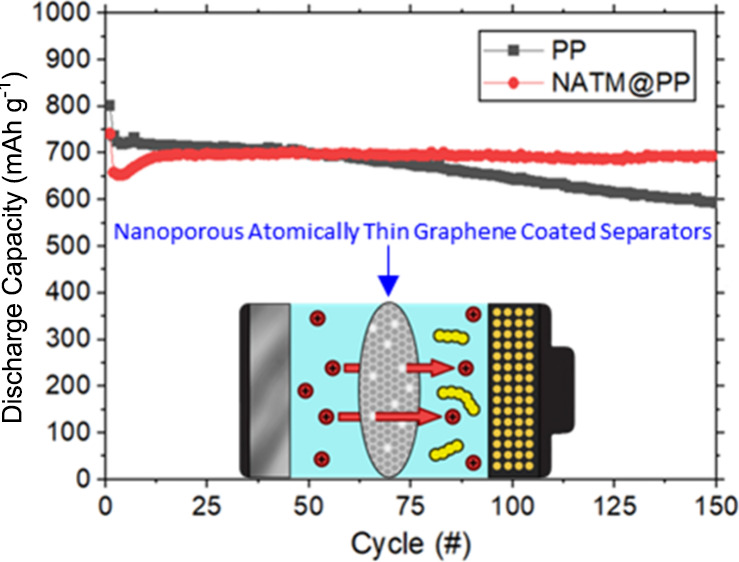

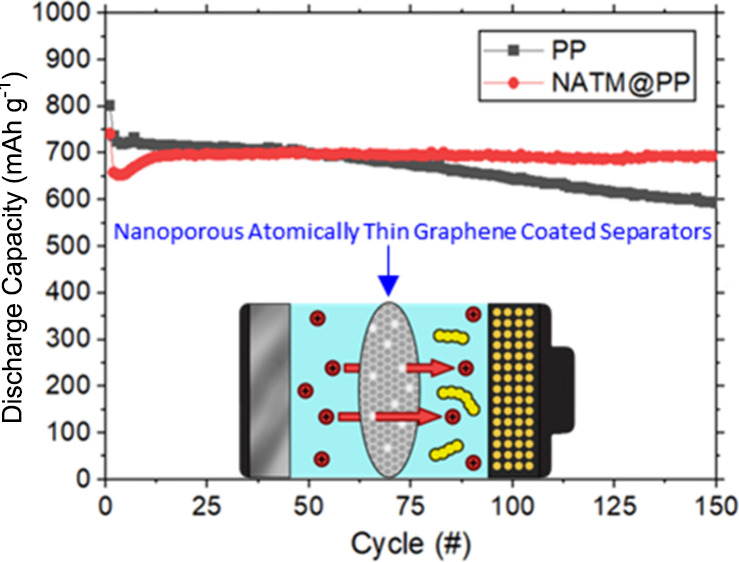

## 1 Introduction

Lithium–sulfur batteries (LSBs) have been a growing topic of interest as researchers look for higher energy density batteries to succeed current lithium-ion batteries (LIBs).^1–3^ LSBs possess a higher theoretical energy density of ∼2600 W h kg^–1^ compared to ∼387 W h kg^–1^ for traditional LIBs due to the high theoretical capacity of sulfur at ∼1672 mA h g^–1^.^3,4^ Obtaining reasonable practical energy densities has unfortunately proven challenging due to the low electronic conductivity of sulfur which limits active material utilization.^5,6^ To increase the electronic conductivity, sulfur is typically composited with conductive carbons at the cost of lower active material ratios.^1,5–9^ However, retaining capacity is also difficult due to the detrimental lithium polysulfide (LiPS) shuttling effect. During discharge, cyclic S_8_ in the cathode is reduced into soluble short-chain LiPS species such as Li_2_S_6_ and Li_2_S_4_. Migration transports the LiPS anions to the anode where they can be irreversibly reduced into insoluble Li_2_S.^1,10,11^ This results in active material loss, poor Coulombic efficiency (CE), and unfavorable Li anode passivation if unmitigated. If these issues are successfully addressed, LSBs have the potential to facilitate electric vehicles with farther ranges and portable electronics with longer periods between recharging. Many strategies have been employed toward this end to regulate LiPS shuttling including tailored electrolytes and modified binders.^3,12–17^ The most widely investigated strategy, however, is likely the use of a modified separator.^18–22^

The separator serves to separate the anode and cathode, preventing an internal short circuit. It can be further modified to provide additional benefits and enhance the battery performance. There are several drawbacks to using modified separators, however, which are not frequently addressed. Modified separators are almost always prepared by casting a powdered solid material and/or polymer onto a traditional polypropylene (PP) separator.^18–22^ This layer can be tens of micrometers thick and contribute significantly to the areal mass of the cell. Modified separator coatings in LSBs can include heterogeneous catalysts which accelerate LiPS conversion kinetics to a great effect but often involve heavy transition metals and difficult to synthesize nanostructured materials.^20,22–24^ Most commonly though, weakly electronegative carbonaceous materials are used.^19,25,26^ However, it is questionable whether demonstrated improvements in kinetics, capacities, and cycle lifetimes are due to electrostatic effects or simply a lower effective sulfur ratio in the cathode. In most studies, improvements are overestimated since the additional carbon coated on the separator is not considered in the electrode active material ratio.^12,25^ Although ion sieving is commonly cited as a LiPS blocking mechanism for LSB separators coated with nanoporous materials, this effect is usually secondary.^24,27,28^ An alternative strategy is to use materials with strong electrostatic/chemical affinities which can bind to dissolved LiPS species and prevent their migration to the anode.^19,28^ However, if this material is not electrically conductive, this will result in active material loss.

Nanoporous atomically thin membranes (NATMs) comprising of monolayer graphene on porous polymer substrates have been shown to enable size-selective ionic and molecular separations.^29–49^ The atomic thinness overcomes hindered diffusion through narrow channels present in commercially available polymeric membranes with tortuous pores.^40^ In this study, a nanoporous atomically thin graphene layer is transferred onto a traditional PP separator (NATM@PP). The tailored nanometer-sized pores in graphene (∼57.3% of pores <0.66 nm, ∼36.1% of pores in the range of ∼0.66–1.5 nm, ∼21.2% of pores >1.5 nm, see Figure S3) hinder transport of bulky LiPS anions (solvated ionic diameters ∼0.81–1.69 nm) to the anode via molecular sieving (Figure 1A,B). In electrochemical cycling studies, the LSB with NATM@PP separator retains ∼94% of initial capacity after 150 cycles compared to ∼74% for unmodified PP demonstrating significant performance improvement. Scanning electron microscopy (SEM) and energy-dispersive X-ray spectroscopy (EDS) on the post-cycled anodes show improved Li morphology and reduced S content, confirming an effective LiPS confinement at the cathode. Compared with most modified separator coatings, nanoporous graphene has the advantage of providing enhanced performance while adding negligible mass or volume to the overall cell. Further, the scalable fabrication process of NATMs used herein provides a facile approach to enable high-performance LSBs.^37,38^

**Figure 1:**
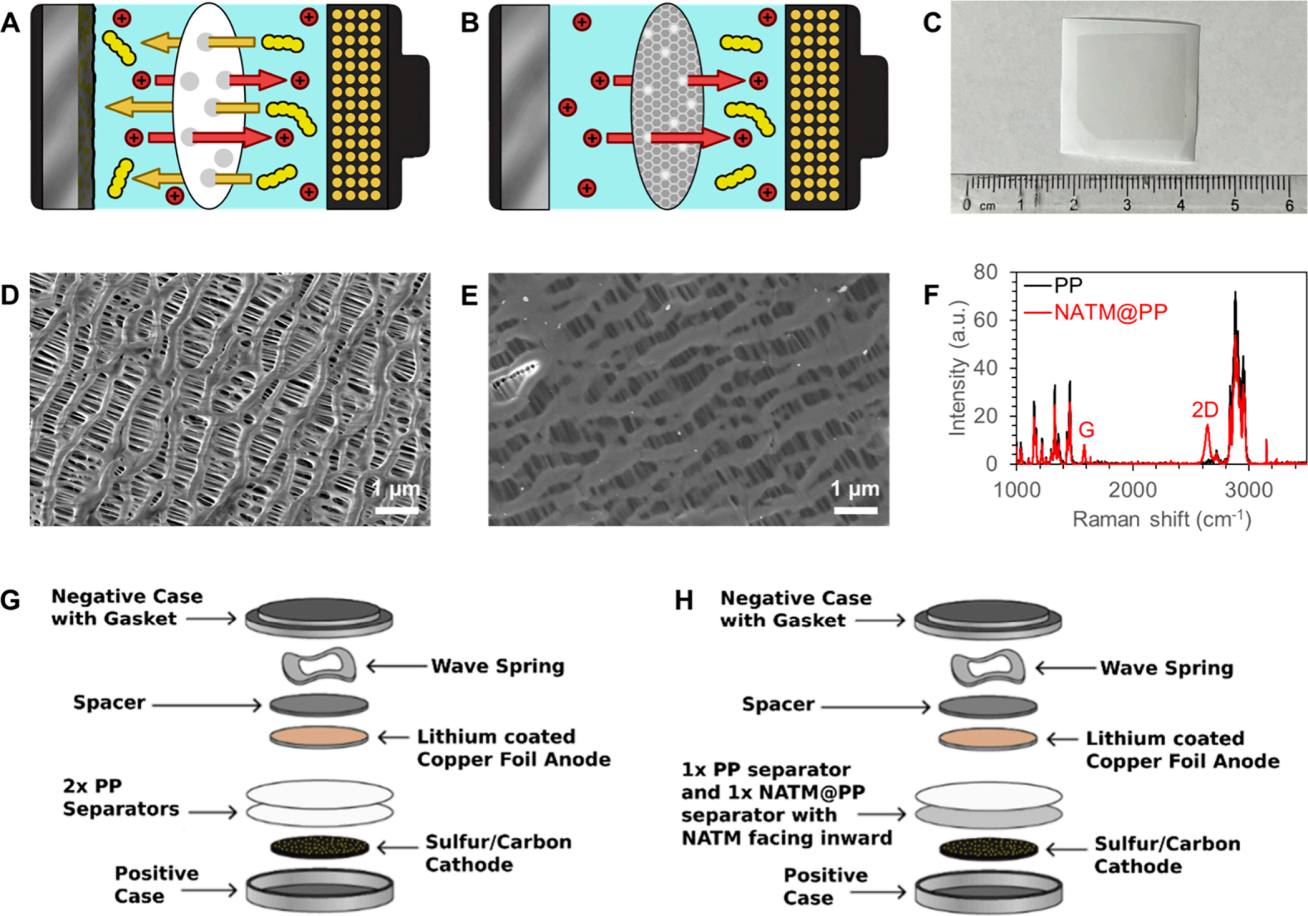
Schematic showing discharge and (A) LiPS shuttling effect for LSBs with PP separators and (B) proposed mechanism for inhibiting LiPS shuttling with nanoporous atomically thin graphene coated on the PP separator. (C) Optical image of prepared NATM@PP separators. The darker region is graphene. SEM images of (D) PP and (E) NATM@PP separators. Bright regions in (E) indicate tear or damage to graphene that results in polymer charging under SEM in those regions. (F) Raman spectra of pristine PP and NATM@PP separators. Schematic of coin cell configuration of LSBs with (G) PP and (H) NATM@PP separators.

## 2 Results and Discussion

### 2.1 Fabrication of NATM@PP Separator

Nanoporous atomically thin graphene was synthesized via facile and scalable low-pressure chemical vapor deposition (CVD)^33,35,37,38^ on Cu foil at a specifically chosen temperature of ∼900 °C based on our prior work that demonstrated the formation of high-density subnanometer scale intrinsic defects (∼0.7–1 nm) in the 2D graphene lattice (also see Figure S3).^33,38^ This pore size was targeted for optimal selectivity based on a study from Babar and Lekakou quantifying the relative solvated ionic diameters of LiPS anions (∼0.81–1.69 nm) and Li^+^ cations (∼0.54–1.26 nm) in DOL:DME solvents.^50^ The tailored pore size would facilitate facile Li^+^ transport, while restricting the permeance of larger LiPS anions. The as-synthesized nanoporous graphene was transferred onto a PP separator using a sacrificial polymer-free isopropanol-assisted hot lamination (IHL) method^36^ to ensure minimal surface contamination^36,38,40,41^ and high transfer yield of >95%.^33,35,36^ We note that the IHL was performed at ∼120 °C, which is lower than the melting point of PP (∼160 °C) to mitigate shrinkage of the PP separator.

Images of the NATM@PP separators can be found in Figure 1C. A dark square of ∼2 × 2 cm indicates the area of the PP, where the atomically thin nanoporous graphene has been transferred. The SEM images of the uncoated PP Celgard 2500 in Figure 1D reveal micron-sized holes with thin connecting fibers formed during the dry-stretch technique.^51,52^ SEM images of NATM@PP (Figure 1E) show high graphene coverage^33,35,36^ (>95%) using roll-to-roll compatible transfer of CVD graphene from the copper CVD substrate to the PP. Most PP pores are covered with suspended graphene, where the darker contrast is due to graphene’s electrical conductivity, while uncovered PP pores appear bright due to polymer charging during SEM imaging. Additional SEM images of uncoated and graphene-coated PP separators can be found in Figures S1 and S2. Atomic resolution transmission electron microscopy images as well as scanning tunneling microscopy of CVD graphene synthesized using identical processes evidenced the presence of nanopores in the graphene lattice,^37,38^ and diffusive-driven transport of ionic and molecular species demonstrates the presence and uniform distribution of nanopores over centimeter-scale areas (Figure S3)^35,38,40^ with ∼42.7% of nanopores <0.66 nm, ∼36.1% of nanopores ∼0.66–1.5 nm, and ∼21.2% of nanopores >1.5 nm.^35,38,40^

Raman spectroscopy in Figure 1F further confirms the successful graphene transfer. In addition to the fingerprint spectra for PP,^53^ the NATM@PP shows peaks corresponding to the G and 2D peaks of graphene at ∼1580 and ∼2640 cm^–1^, respectively.^38,54^ Raman spectra support the presence of defects in the graphene lattice that manifest as nanopores via the presence of a D peak consistent with prior reports (see Figure S4).^38,54^ Additional spectra of the nanoporous and defect-free high-quality graphene (HQGr) are represented in Figure S4.

### 2.2 Electrochemical Characterization

Electrochemical testing of LSBs was performed in coin cells with a lithium anode, sulfur/carbon composite cathode, and standard 1:1 v/v DOL:DME electrolyte with 1.0 M LiTFSI salt and 0.3 M LiNO_3_ to aid in anode passivation. Schematics of the coin cell configuration can be found in Figure 1G,H. To avoid exfoliation, abrasion, or other damage of the NATM which could occur if left in contact with the electrode due to volumetric changes or electrochemical reactions, an additional separator is used to sandwich the NATM between two layers of PP. Future studies could use two thinner separators. However, for a comparable control, two layers of PP are also used in the standard LSB without graphene. The separators are sized at 19 mm in diameter so that the CR2032 negative cap gasket forms a complete seal between electrodes and prevents electrolyte leakage around the separators which would undermine the function of the selective NATM layer.

Galvanostatic cycling data of the discharge capacity and CE for LSBs with PP and NATM@PP separators are represented in Figure 2A,B, respectively. Overall, the LSB with the NATM@PP separator shows greatly improved capacity retention, demonstrating effectiveness of the NATM in preventing sulfur loss (see details below). Since LSBs are constructed in the charged state, CE = charge capacity/discharge capacity. Cells show a capacity drop and low initial CE as LiPSs saturate the electrolyte during first discharge, where sulfur dissolution is the greatest.^55^ During charging, solubilized LiPSs, which are generated spontaneously due to self-discharge or from the normal discharge process, are deposited back into the cathode as they are oxidized back into insoluble S_8_.^56,57^ The lower initial CE of NATM@PP (97.4% vs 99.2%) indicates that the NATM@PP is effective in mitigating the overcharge behavior of LSBs due to spontaneous discharge caused by polysulfide shuttling. Also, in contrast to PP with gradual capacity loss over cycles 2–15 due to dissolved sulfur being lost to anode passivation, NATM@PP shows a capacity rise as sulfur dissolves and more evenly redeposits on conductive carbon surfaces in the cathode, effectively increasing the sulfur utilization. This is reflected in the higher CE for NATM@PP over the same period, as the overcharging reflects the reutilization of dissolved LiPS.^56–58^ It is worth noting that the current due to self-discharge is not directly measured since the electron transfer between the lithium and sulfur occurs internally, but reoxidation may be reflected in the charge process. Similarly, passivation is not reflected in the discharge, as this is another internal electron transfer, but the absence of this active material may be reflected in the charge step. Overall, average CE’s for PP (control) and NATM@PP are very similar at ∼101.3% and ∼101.4% over cycles 1–15, respectively.

**Figure 2:**
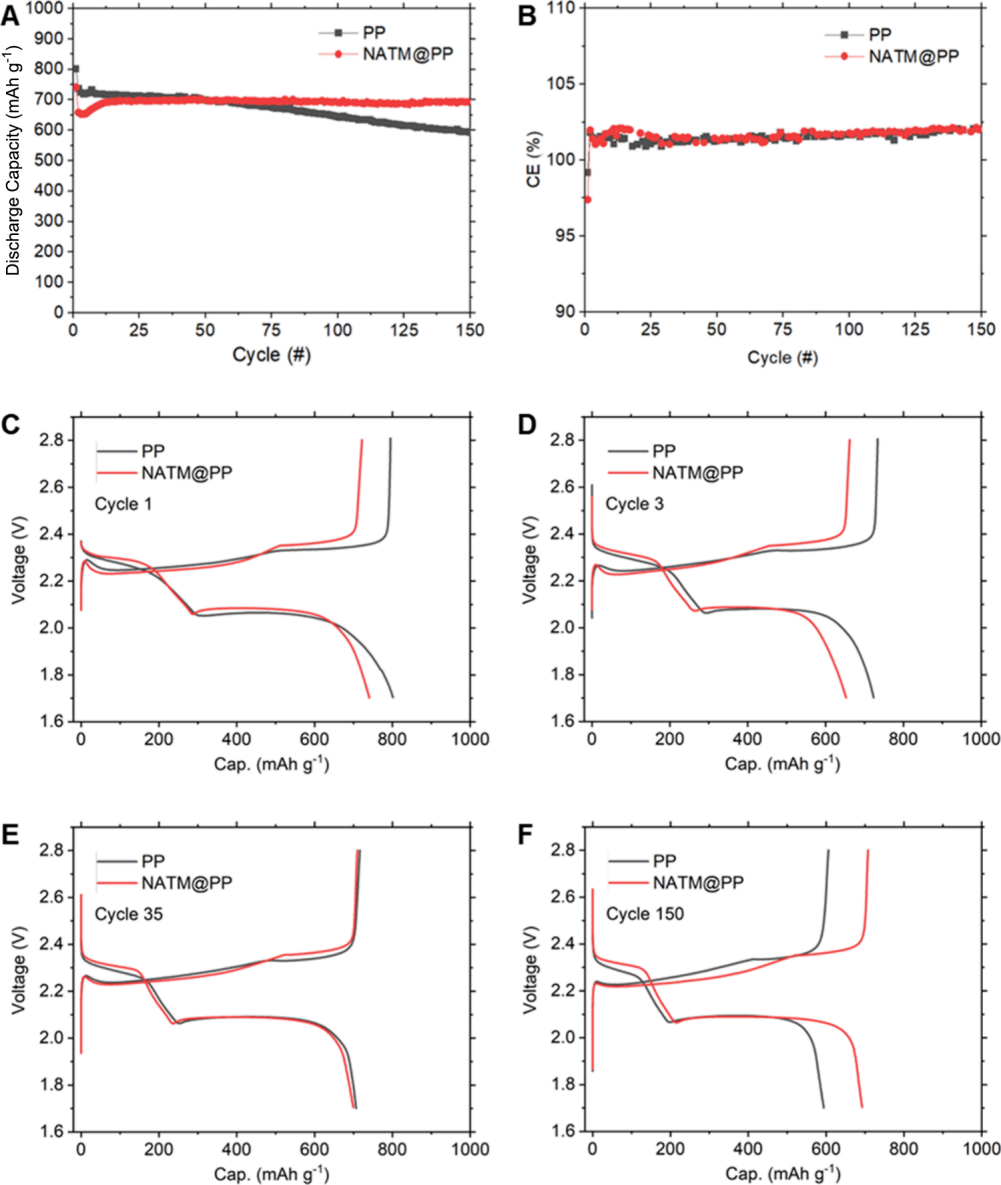
Galvanostatic C/5 cycling of prepared LSBs showing (A) discharge capacity and (B) CE as a function of number of cycles with select voltage profiles for numbers (C) 1, (D) 3, (E) 35, and (F) 150. Also see Figure S5.

From cycles 15–150, NATM@PP shows remarkable capacity retention, with virtually no capacity loss (695 vs 694 mA h g^–1^). In comparison, PP retains 83.1% of its capacity over the same period, declining from 716 to 592 mA h g^–1^. The capacity of NATM@PP approaches and eventually surpasses that of standard PP (control) due to effective sulfur confinement. After 150 cycles, however, capacity eventually fades for NATM@PP, likely due to damage sustained to the fragile NATM from cyclic volume changes (Figure S6). Damage to the NATM is discussed later in the postcycling analysis. Overall, after 300 cycles, PP retains ∼66.7% of its initial capacity and has an average CE of ∼102.0%, compared to ∼79.8 and ∼102.2% for NATM@PP, respectively (Figure S6).

Select voltage profiles for cycles 1, 3, 35, and 150 are listed in Figure 2C–F. Additional profiles are listed in Figure S5. Throughout cycling, the LSB with the NATM@PP separator shows a narrower voltage hysteresis, suggesting a reduced Li_2_S passivation and consequently lower impedance at the anode.^59^ In cycle 1, the drop-off from the low voltage plateau corresponding to the final precipitation of soluble Li_2_S_2_ into Li_2_S is much narrower due to an effective sulfur confinement in the cathode and limiting of the continuous discharge and dissolution of sulfur into the electrolyte. Over continued cycling, the voltage drop-off becomes steeper for both.^1,15,60^

A HQGr (devoid of a high density of nanopores)-coated separator (HQGr@PP)^31,33,34,36–38,40,41^ was also tested to highlight the importance of the tailored nanopore size (Figure S6). The HQGr film used here is similarly a monolayer graphene sheet with atomic-scale thickness and negligible mass loading and is not expected to introduce bulk transport barriers due to excessive deposition. The capacity for HQGr@PP starts low due to steric resistance of the nonporous carbon atomic monolayer impeding transport of Li^+^ but gradually increases, likely due to similar exfoliation. The reduced performance is therefore attributed to the intrinsic impermeability of pristine graphene, which lacks the nanoscale pathways necessary for efficient Li^+^ transport. However, without the molecular sieving effect of the nanopores, the HQGr@PP separator subsequently also shows rapid capacity fading. Detailed characterization of HQGr can be found in prior publications.^31,33,34,36–38,40,41^

The impact of NATM on electrolyte resistance and anode passivation is investigated through electrochemical impedance spectroscopy (EIS). The Nyquist plots of LSBs before and after the long-cycling study are shown in Figure 3A,B, respectively. Here, the bulk resistance (*R*_B_), SEI resistance (*R*_SEI_), and charge-transfer resistance (*R*_CT_) are correlated to the x-intercept, first semicircle, and second semicircle, respectively.^59^ These parameters are then extracted by performing a Z-fit of the equivalent circuit in Figure S7 and represented in Table 1. A complete set of fitting parameters can be found in Table S1. Before cycling, cells with both separators show similar *R*_B_ due to the low energy barrier of Li^+^ crossing the NATM. Similarly, since SEI has not been formed prior to cycling, *R*_SEI_ is effectively unchanged as well. Although *R*_CT_ appears larger, this should similarly be unchanged and may reflect difficulty in fitting the semicircle, which has become convoluted with the large low-frequency tail due to capacitive effects from the NATM interlayer. After cycling, effective LiPS confinement for NATM@PP results in more favorable anode passivation and a reduced *R*_SEI_. However, over the course of many cycles, *R*_B_ has comparatively increased, potentially due to a higher polysulfide concentration in the electrolyte or clogging of nanopores through sulfur disproportionation. The low-frequency constant phase element tail also diminishes in intensity, further suggesting damage may have been sustained to the fragile NATM over many cycles.

**Figure 3:**
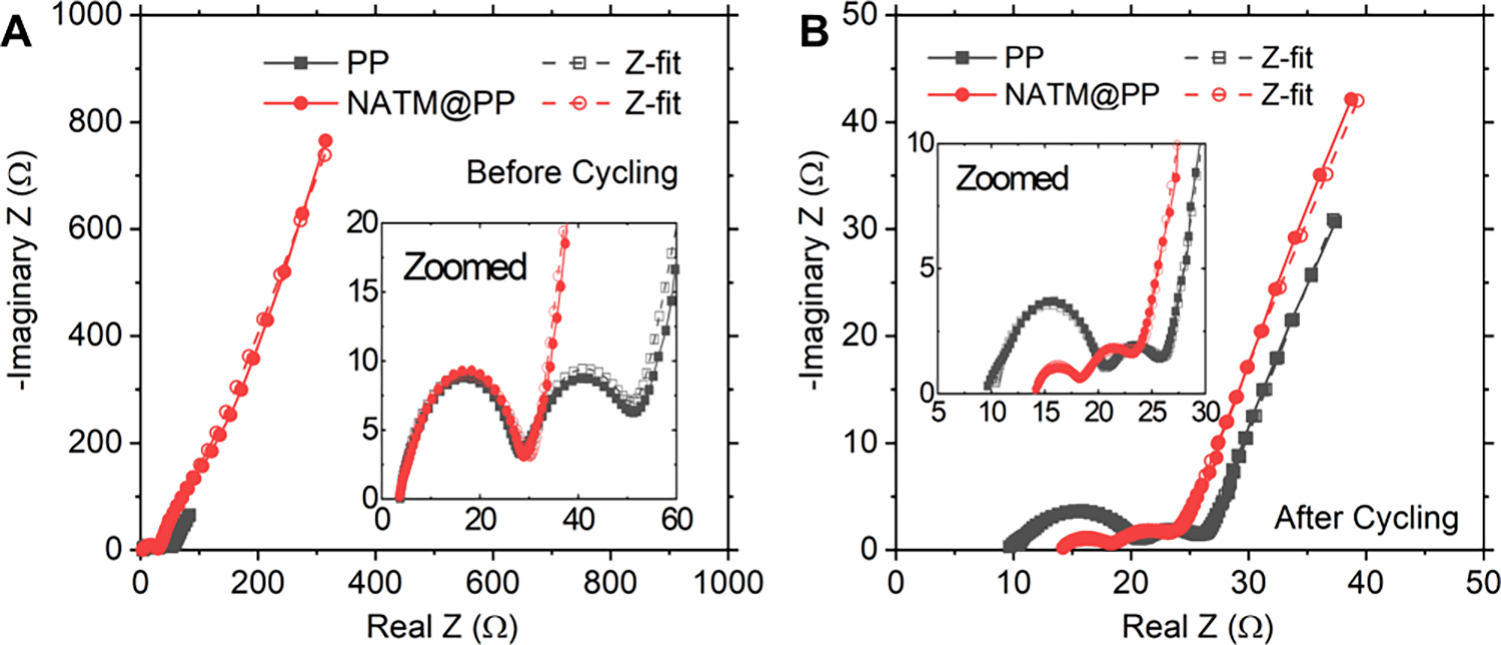
Nyquist plots of EIS spectra for LSBs (A) before and (B) after cycling.

**Table 1:** Resistance Values Obtained from Z-Fits to the EIS Spectra in Figure 3

		*R*_B_ [Ω]	*R*_SEI_ [Ω]	*R*_CT_ [Ω]
before	PP	3.65	26.04	20.75
	NATM@PP	3.58	26.71	35.13
after	PP	10.16	10.24	6.68
	NATM@PP	14.01	4.25	7.17

A rate-study was performed to investigate if the NATM would add any additional impedances which could impact kinetics (Figure 4). Overall, the NATM@PP separator does not appear to impact rate capabilities in any significant way and performs similarly to the standard PP separator (Figure 4A). As with the long cycling study, CE is improved with NATM@PP at all C rates, supporting the LiPS blocking mechanism (Figure 4B). Additionally, it can be observed that the CE for both separators becomes closer to 100% as C rate increases since the time for LiPSs to form and self-discharge shortens.^57^

**Figure 4:**
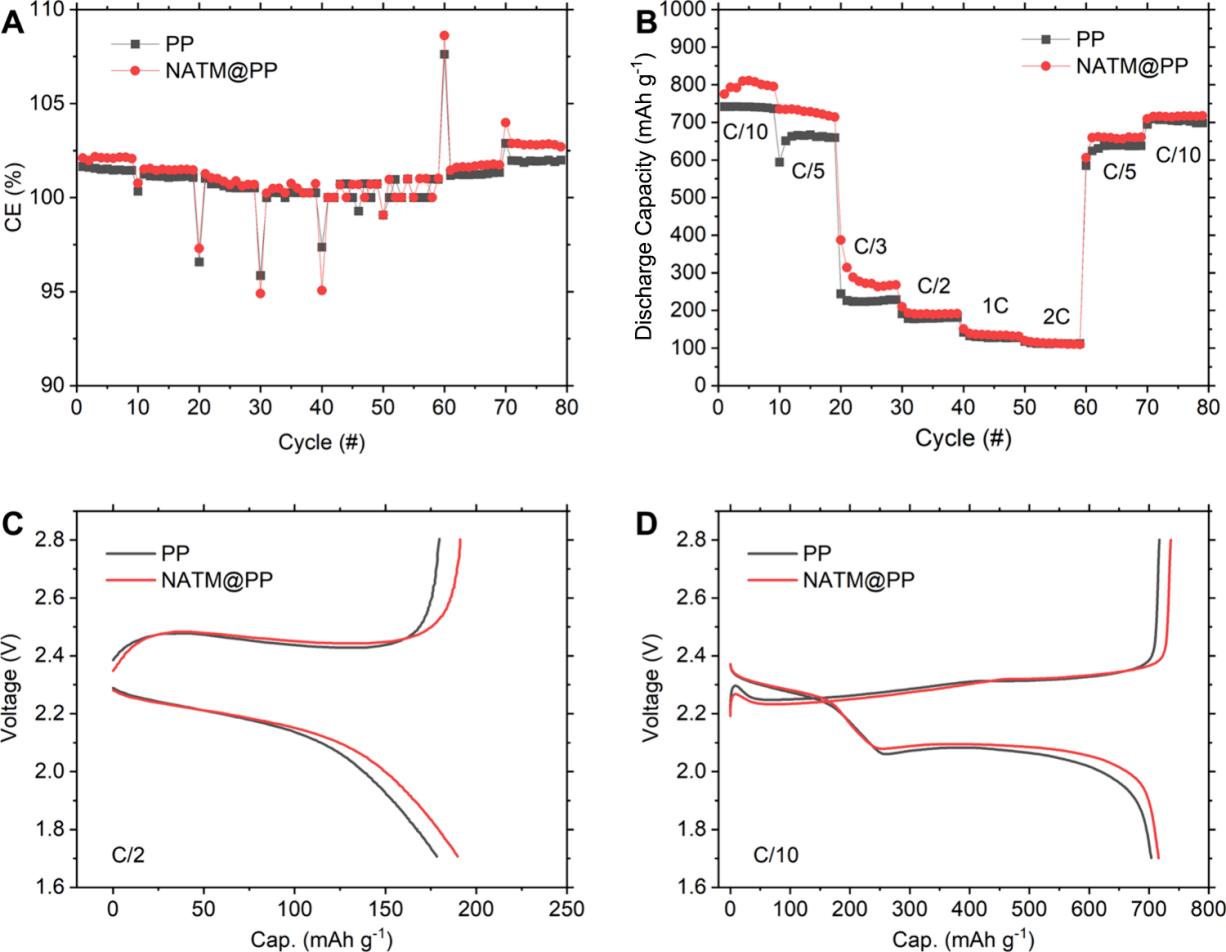
Rate-study cycle showing (A) discharge capacity and (B) CE versus cycle number of prepared LSBs with selected voltage profiles for (C) C/2 and (D) C/10 rates, taken from cycles 40 and 80, respectively.

### 2.3 Postcycling Characterization

After cycling, LSBs were disassembled for SEM imaging, as shown in Figure 5. Postcycled PP and NATM@PP separators are shown in Figure 5A,B, respectively. The PP separator appears unchanged. However, NATM@PP shows signs of graphene exfoliation or damage, evidenced by the bright regions charging the uncovered polymer substrate, where conductive graphene is no longer present. This minor level of exfoliation helps explain the eventual decline in the capacity observed in the long-cycling study. Further, we note that some exfoliation could also occur during disassembly.

**Figure 5:**
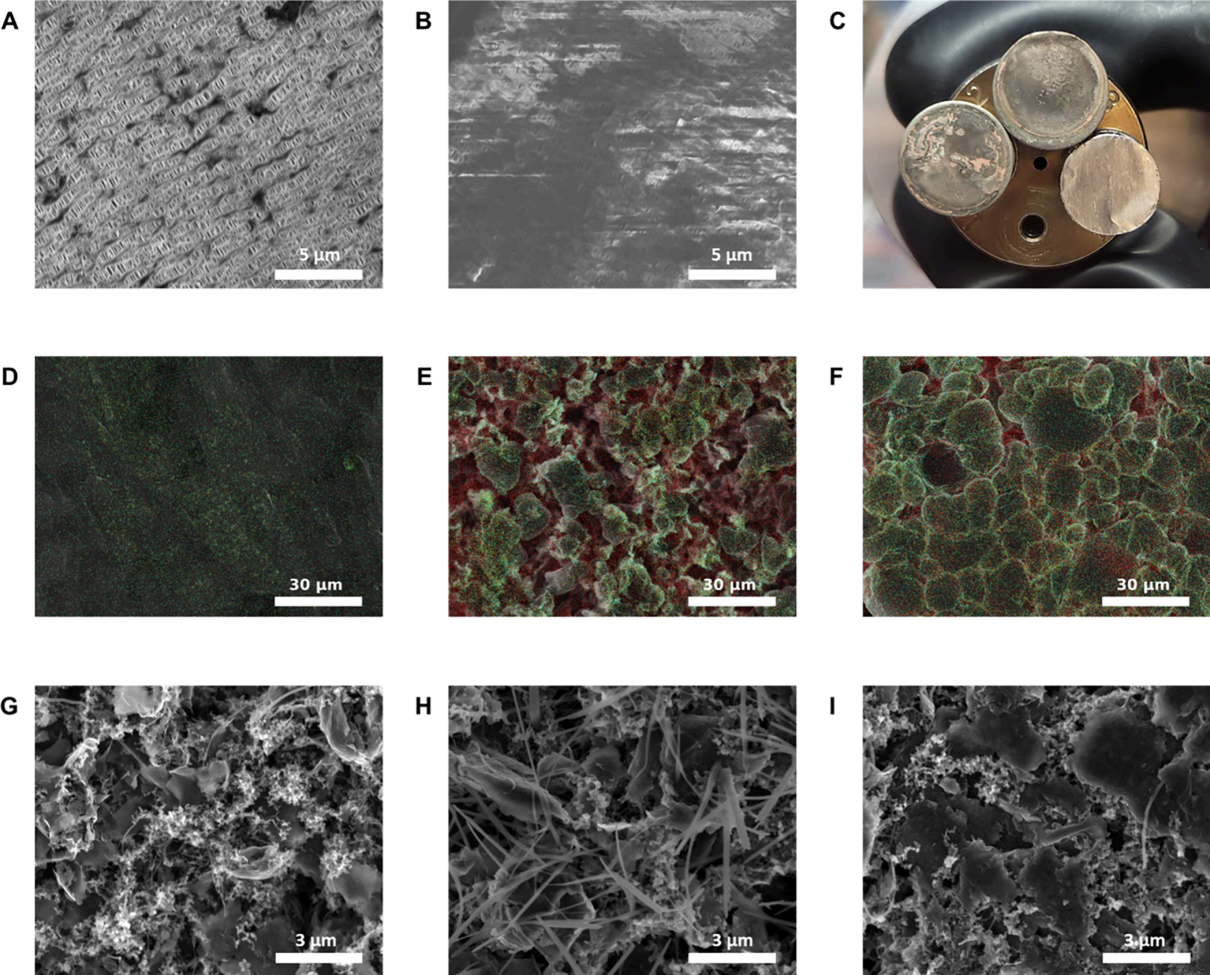
SEM images of postcycled (A) PP and (B) Gr@PP separators. (C) Optical images of Li anodes of postcycled LSBs. SEM images with overlaid EDS elemental mapping of Li anodes from LSBs (D) before and (E) after cycling and (F) Gr@PP separators after cycling. Elements represented in EDS elemental mappings include S (red), N (yellow), O (green), and F (cyan). SEM images of (G) pristine cathode and postcycled cathode for LSBs with (H) PP and (I) Gr@PP separators. Also see Figures S8–S13.

The anode of the prepared LSBs was imaged with SEM and EDS analyses. Photographs of pristine and postcycled Li anodes in Figure 5C show poor and uneven Li plating with the PP separator. The NATM@PP anode on the other hand shows more even Li plating and no bare copper.^61^ SEM/EDS further probes the SEI composition and Li-plating morphology, as shown in Figure 5D–F, before and after cycling for the PP and NATM@PP separators, respectively. Compared to the smooth Li (Figure 5D) surface before cycling, the cycled PP anode in Figure 5E shows a mixture of needle-like and globular Li. The needle-like Li dendrites form because of poor plating due to the unfavorable and resistive Li_2_S passivation, as evidenced by the red EDS mapping corresponding to S.^61–63^ In comparison, the NATM@PP anode shows a good plating morphology with large, densely packed Li globules (Figure 5F). The elemental EDS mapping reveals a much higher portion of area colored by a mix of yellow, green, and cyan, indicating the presence of Li_3_N, organic oxides, and LiF due to the reduction of LiNO_3_, DOL:DME, and LiTFSI, respectively.^57,58,61–63^ This is supported by quantitative EDS analysis in Table 2. Passivation layers rich in these compounds are known to facilitate ionically conductive and robust SEI on Li metal, explaining the enhanced morphology.^61–63^ Additional SEM images and EDS spectra of cycled Li anodes can be found in Figures S8–S11. These postcycling characterizations indicate that the observed performance improvements could be attributed to LiPS blocking by the NATMs. The observed improved passivation is consistent with observations from electrochemical studies and helps elucidate the causes of lower SEI resistances and improved capacity retention.

**Table 2:** EDS Quantitative Elemental Analysis Values for SEM/EDS Images Are from Figure 5

	EDS quantitative wt %						
	C	N	O	F	S	Cu	total
pristine	25.04	0.48	73.57	0.26	0.65	ND	100.00
PP	7.96	4.46	46.09	26.22	15.27	ND	100.00
NATM@PP	8.55	4.36	44.90	32.93	9.26	ND	100.00

Finally, SEM images of before and after cycling of PP and NATM@PP cathodes are represented in Figure 5G–I. In comparison to the NATM@PP cathode, which looks similar to the pristine, the cycled PP cathode does not appear densely packed. Evidence of sulfur loss is present, with the bare scaffold of carbon underneath clearly visible. Additional SEM images of separators and cathodes are available in Figures S12 and S13.

## 3 Conclusions

Our study demonstrates a novel application of nanoporous atomically thin membranes (NATMs) in Li–S batteries. To the best of our knowledge, this is one of the first studies to investigate the molecular sieving with NATM as a primary mechanism to mitigate LiPS shuttling. The NATM@PP separator with tailored pore size effectively suppresses the LiPS shuttling effect by blocking large LiPS anions, resulting in greater capacity retention due to reduced sulfur loss to anode passivation. Notably, NATM@PP facilitates virtually no capacity loss over 150 cycles. The atomically thin nature of the graphene coating means that these benefits can be obtained without introducing significant impedances to Li^+^ permeation or adding extra bulk, as with most carbon-coated separators. Performance may potentially be further improved by utilizing a thermally stable polyamide-based substrate with submicron sized pores in place of PP. This could address the identified issues related to delamination by enabling graphene transfer to a smoother porous substrate at higher temperatures. Future studies may also involve interfacial polymerization techniques^64^ to further tune the pore density and reduce LiPS leakage through larger defects as well as techniques to improve adhesion of the NATM to the PP separator to mitigate potential delamination from volumetric changes during cell cycling and improving capacity retention.

## 4 Materials and Methods

### 4.1 Graphene Growth

Nanoporous monolayer graphene was synthesized via the low-pressure chemical vapor deposition (LPCVD) method as described in detail elsewhere.^33,35,37,38^ Initially, ∼2 × 7 cm^2^ polycrystalline Cu foil (99.9% purity, 18 μm thickness, JX Holding HA) was precleaned via sonication in diluted nitric acid (20%) for 4 min (2 min per side), followed by thorough rinsing in DI water and drying in air. Next, the Cu foil was annealed in a 1 in. hot-walled tube furnace (Thermo Scientific Lindberg/Blue M Mini-Mite) at 1060 °C for 30 min under 100 sccm H_2_ (system pressure ∼4 Torr). After that, the system was cooled to 900 °C (nanoporous graphene growth temperature) in 10 min under 60 sccm H_2_ (∼1 Torr) and maintained for 20 min. Nanoporous graphene was then grown by adding 3.5 sccm of CH_4_ (∼0.9 Torr) for 30 min and 7 sccm of CH_4_ (∼0.85 Torr) for 30 min to H_2_ (60 sccm). Finally, the Cu foil was quench-cooled to room temperature in a growth atmosphere (7 sccm CH_4_ and 60 sccm H_2_).

HQGr^31,33,34,36–38,40,41^ was grown using the same system and Cu foil. The Cu foil was first annealed at 1060 °C under 100 sccm of H_2_ (∼4 Torr) for 60 min and then under 300 sccm of H_2_ (∼14 Torr) for 15 min. After annealing, graphene growth was achieved by adding 0.5 sccm of CH_4_ (∼12 Torr) for 45 min and 1 sccm of CH_4_ (∼12 Torr) for 30 min to the system atmosphere. Finally, the Cu foil was quench-cooled to room temperature in a growth atmosphere (1 sccm CH_4_ and 300 sccm H_2_).

### 4.2 Graphene Transfer to PP Separator

Graphene was transferred to a PP separator (Celgard 2500, 55% porosity, 25 μm thick) using the IHL method.^36^ The bottom layer of graphene was first pre-etched in ammonium persulfate solution (0.2 M) for 15 min, followed by rinsing in DI water and drying in air.^31–38,40,41^ Next, the PP separator was placed against the pre-etched graphene/Cu foil with the graphene side facing up and then sandwiched between two pieces of weighing paper to build a paper/PP/graphene/Cu/paper stack. After adding a drop of isopropyl alcohol (∼100 μL) to the PP/graphene interface, the stack was laminated with Teflon protective layers at 120 °C using an office laminator (TruLam TL-320E). After peeling off the top weighing paper, the PP/graphene/Cu stack was gently floated on the APS solution (0.2 M) to completely etch the Cu foil. Finally, the PP/graphene stack (NATM@PP) was rinsed with DI water, washed with ethanol, and dried in air.

### 4.3 Materials Characterizations

SEM imaging, EDS mapping, and quantitative analyses are performed using a JEOL 600+ benchtop unit. Higher resolution field-emission SEM images of pristine separators are obtained using a Zeiss Merlin Scanning Electron Microscope with a Gemini II Column operated at 2 kV, and postcycled cathodes and separators were imaged using a Nova Nano SEM 200 at 3 kV. Raman spectra were gathered via a Thermo Scientific DXR Raman microscope with 633 nm laser.

### 4.4 Electrode Fabrication and Cell Preparation

Sulfur/carbon composite was first prepared by ball-milling (20 Hz, 30 min) 2 g of sulfur (<55 nm, 99.99%, SkySpring Materials) with 1 g nanopowder multilayer flakes (Graphene Supermarket, AO-4). A slurry is then prepared by combining S/C composite, carbon nanotubes, Super P carbon black (Timcal), and PVDF (Kynar HSV-900) at 75:5:5:15 weight ratios with an appropriate amount of NMP solvent in a planetary mixer (Thinky) for 30 min. The slurry is then cast onto carbon-coated Al foil (MSE) and dried for 12 h at 45 °C. Resulting S loading is ∼1.5 mg cm^–2^. Electrodes and PP separators (Celgard 2500) are then punched into 14 and 19 mm disks, respectively, and transferred to a glovebox (Vac) for cell assembly. NATM@PP separators are carefully cut to 19 mm using a razor blade to avoid delamination. Under argon atmosphere, Li anodes are punched to 14 mm from lithium-coated copper foil (Honjo Metal, 99.9%). Electrolyte was prepared with 1:1 v/v 1,3 dioxolane (DOL, Aldrich, anhydrous 99.8%) and 1,2-dimethoxyethane (DME, Aldrich, anhydrous 99.5%) with 1.0 M bis­(trifluoromethane)­sulfonimide lithium salt (LiTFSI, Aldrich, 99.99%) and 0.3 M LiNO3 additive (Alfa Aesar, 99%). Salts were dried in a vacuum oven at 120 °C for 3 days. Cell components and electrolyte are then layered (Figure 1G,H) in Cr2032 stainless-steel cell parts (MTI) and crimped. Resulting E/S ratio is ∼19. Two PP separators are used in each cell, with the NATM being insulated between two layers of PP.

### 4.5 Electrochemical Characterizations

Galvanostatic cycling is performed using an MTI BST8–3 Battery Analyzer, and EIS spectra were recorded with a Gamry Reference 600+ potentiostat. Z-fits were performed by using EC Lab software.

## Supplementary Material



## Notes

Figure S1: pristine PP separator at ×10,000 magnification; Figure S2: pristine NATM@PP separator at (a) ×10,000 and (b) ×50,000 magnification; Figure S3: (A) diffusive permeance and (B) normalized diffusive flux of 900 °C graphene and HQGr transferred onto PCTE support for KCl and Vitamin B12; Figure S4: Raman spectra of substrate-free NATM and HQGr; Figure S5: additional select voltage profiles from C/5 cycling in Figure 3 at cycles (A) 10 and (B) 275; Figure S6: HQGr@PP cycling showing (a) discharge capacity and (b) CE versus cycle number; Figure S7: equivalent circuit used in Z-fits to Nyquist plots; Figure S8: additional SEM images of pristine Li anode; Figure S9: additional SEM images of postcycled Li anode with PP separator; Figure S10: additional SEM images of postcycled Li anode with NATM@PP separator; Figure S11: EDS spectra of pristine lithium anode as well as postcycled lithium anodes with PP and NATM@PP separators; Figure S12: additional FESEM photos pf postcycled PP and NATM@PP separators; Figure S13: additional FESEM images of pristine and postcycled cathodes with PP and NATM@PP separators; Table S1: summary of all Z-fit parameters from Figure 3 (PDF)
