# Supplementary material for: Size-Selective Nanoporous Atomically Thin Graphene Separators for Lithium–Sulfur Batteries
Source: ACS Appl Mater Interfaces. 2025 Sep 4;17(39):55554–63. doi: 10.1021/acsami.5c11148 (PMC12492388; doi:10.1021/acsami.5c11148)
Supplement: Supplementary file 1 [file am5c11148_si_001.pdf]

Supporting Information

Size Selective Nanoporous Atomically Thin Graphene Separators for Lithium Sulfur Batteries

Authors: *Daniel A. Gribble,* [†§] *Peifu Cheng*[‡§] *Vilas G. Pol,** [†] *Piran R. Kidambi**[,^]

[†]Davidson School of Chemical Engineering, Purdue University, West Lafayette, Indiana 47907, United States.

[‡]Department of Chemical and Biomolecular Engineering, Vanderbilt University, Nashville, Tennessee 37212, United States.

^Department of Mechanical and Aerospace Engineering, University of Florida, Gainesville, Florida 32611, United States.

[§]These authors contributed equally.

*E-mail: vpol@purdue.edu; p.kidambi@ufl.edu

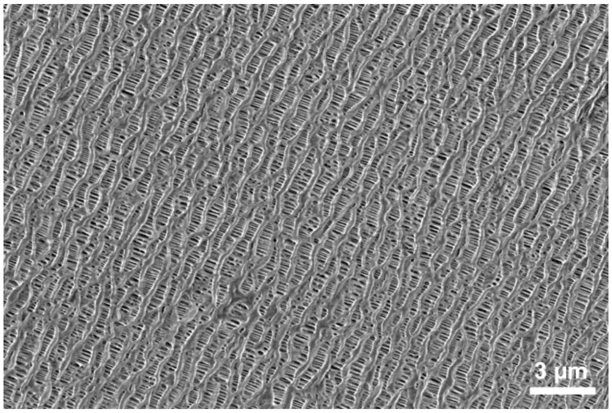

Figure S1. SEM image of PP separator at 10,000× magnification.

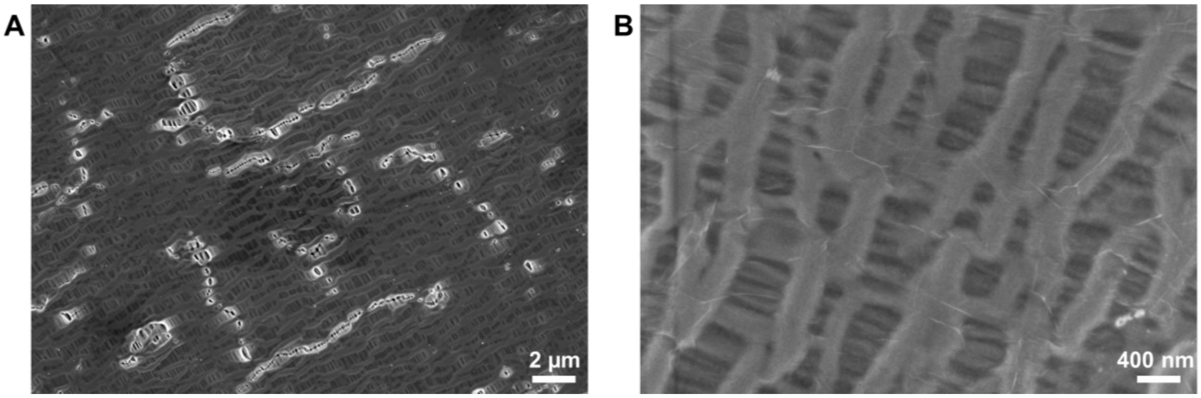

Figure S2. SEM image of NATM@PP separator at A) 10,000× and B) 50,000× magnification.

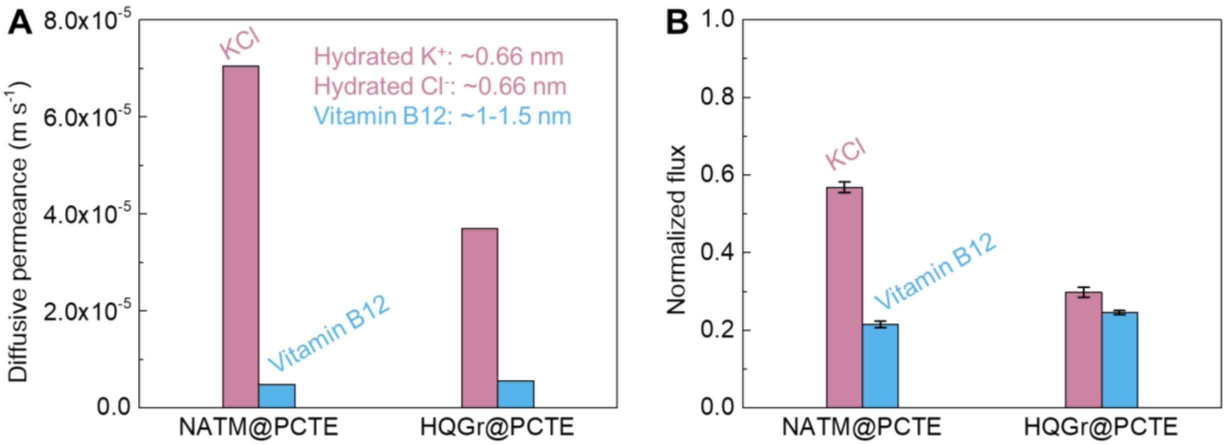

Figure S3. A) Diffusive permeance and B) Normalized diffusive flux of ~

900 °C graphene transferred onto PCTE support (NATM@PCTE) and high-quality graphene transferred onto PCTE support (HQGr@PCTE) for KCl (hydrated diameter of ions ~0.66 nm) and Vitamin B12 (diameter ~1−1.5 nm). The difference between the normalized flux of KCl ~57.3% and B12 ~21.3% for NATM@PCTE indicates the presence of defects between ~0.66 to 1−1.5 nm. Reproduced with the permission from Ref. 33, Copyright 2022, American Chemical Society.

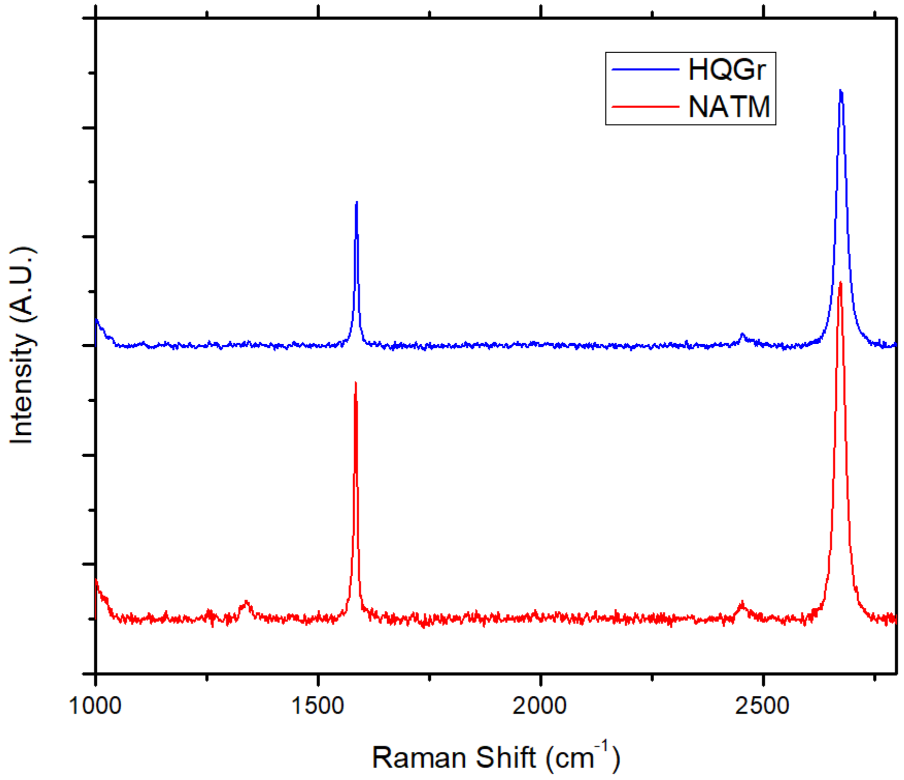

Figure S4. Raman spectra of graphene with nanopores synthesized at ~900°C (NATM) and high-quality graphene (HQGr) after transfer to 300 nm SiO_2/Si wafer.

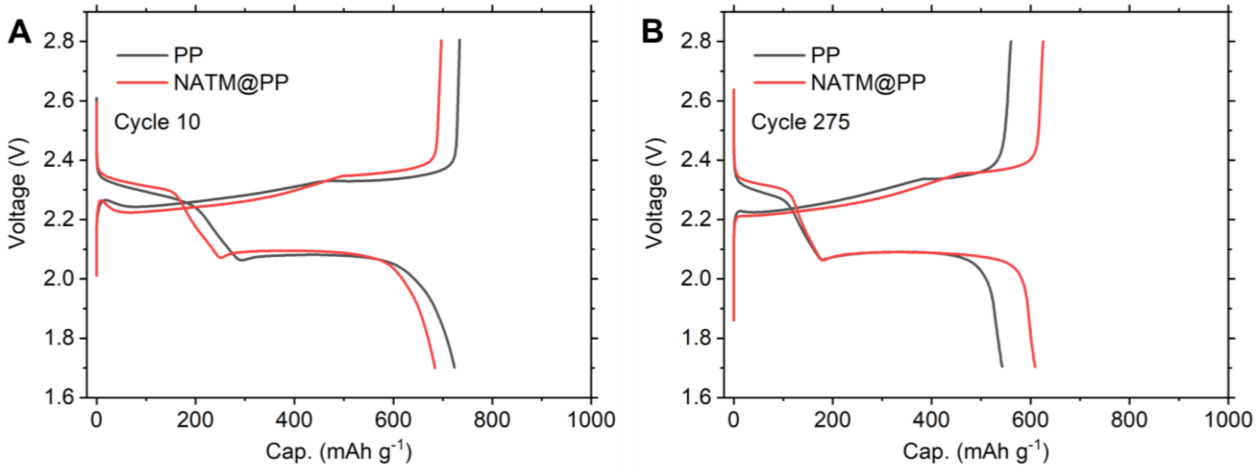

Figure S5. Additional select voltage profiles from C/5 cycling in Figure 3 at cycles (A) 10 and (B) 275.

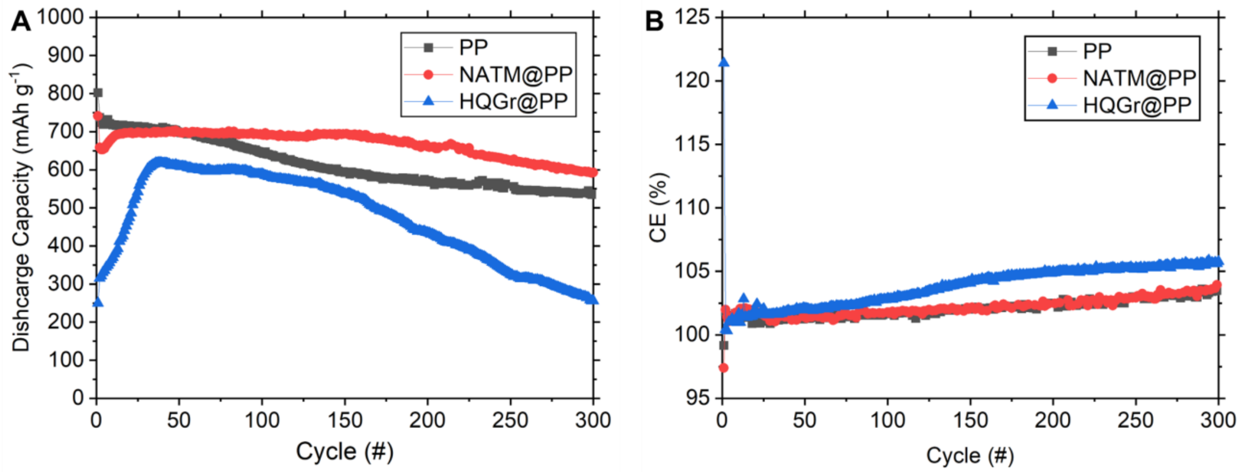

Figure S6. High-quality graphene on PP separator (HQGr@PP) cycling showing A) discharge capacity and B) CE versus cycle number.

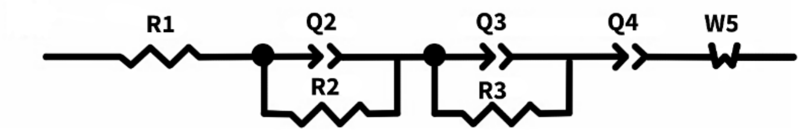

Figure S7. Equivalent circuit used in Z-fits to Nyquist plots.

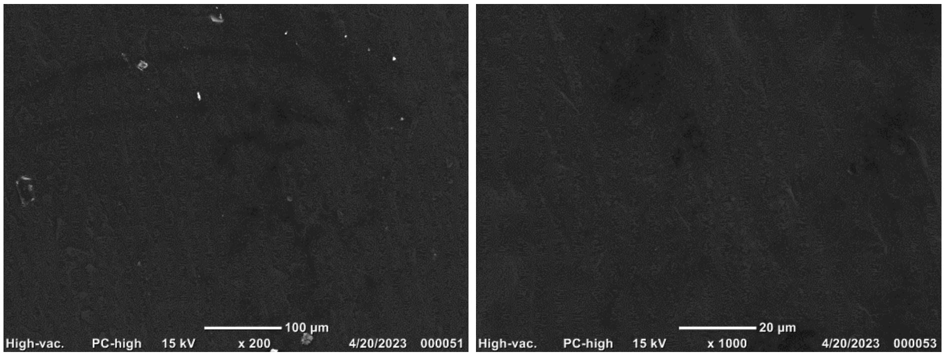

Figure S8. Additional SEM images of pristine Li anode at 200× (left) and 1000× (right) magnification.

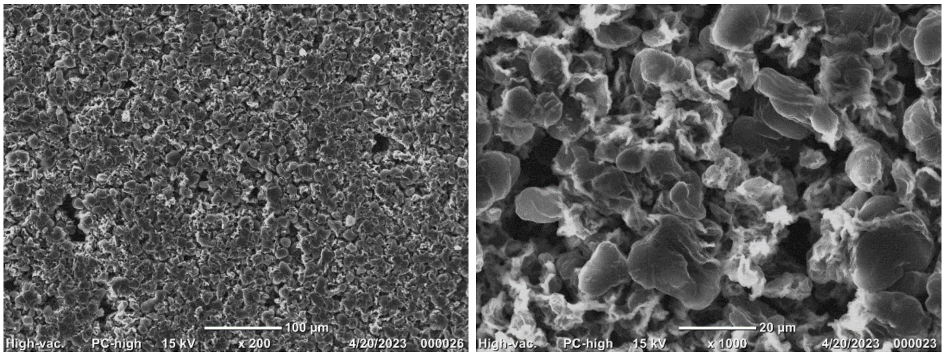

Figure S9. Additional SEM images of post-cycled Li anode with PP separator at 200× (left) and 1000× (right) magnification.

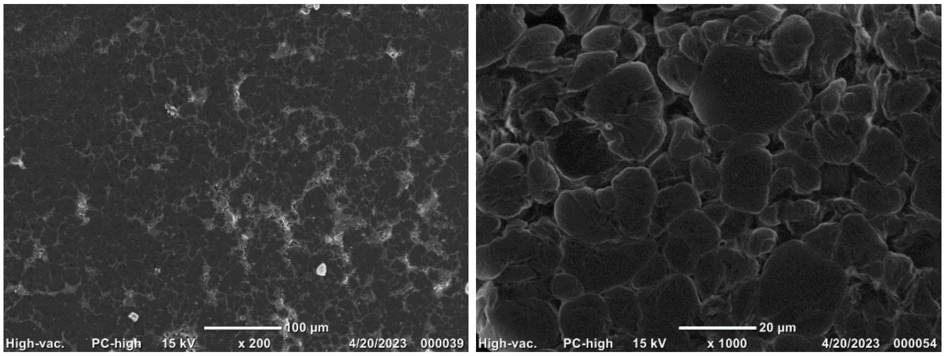

Figure S10. Additional SEM images of post-cycled Li anode with NATM@PP separator at 200× (left) and 1000× (right) magnification.

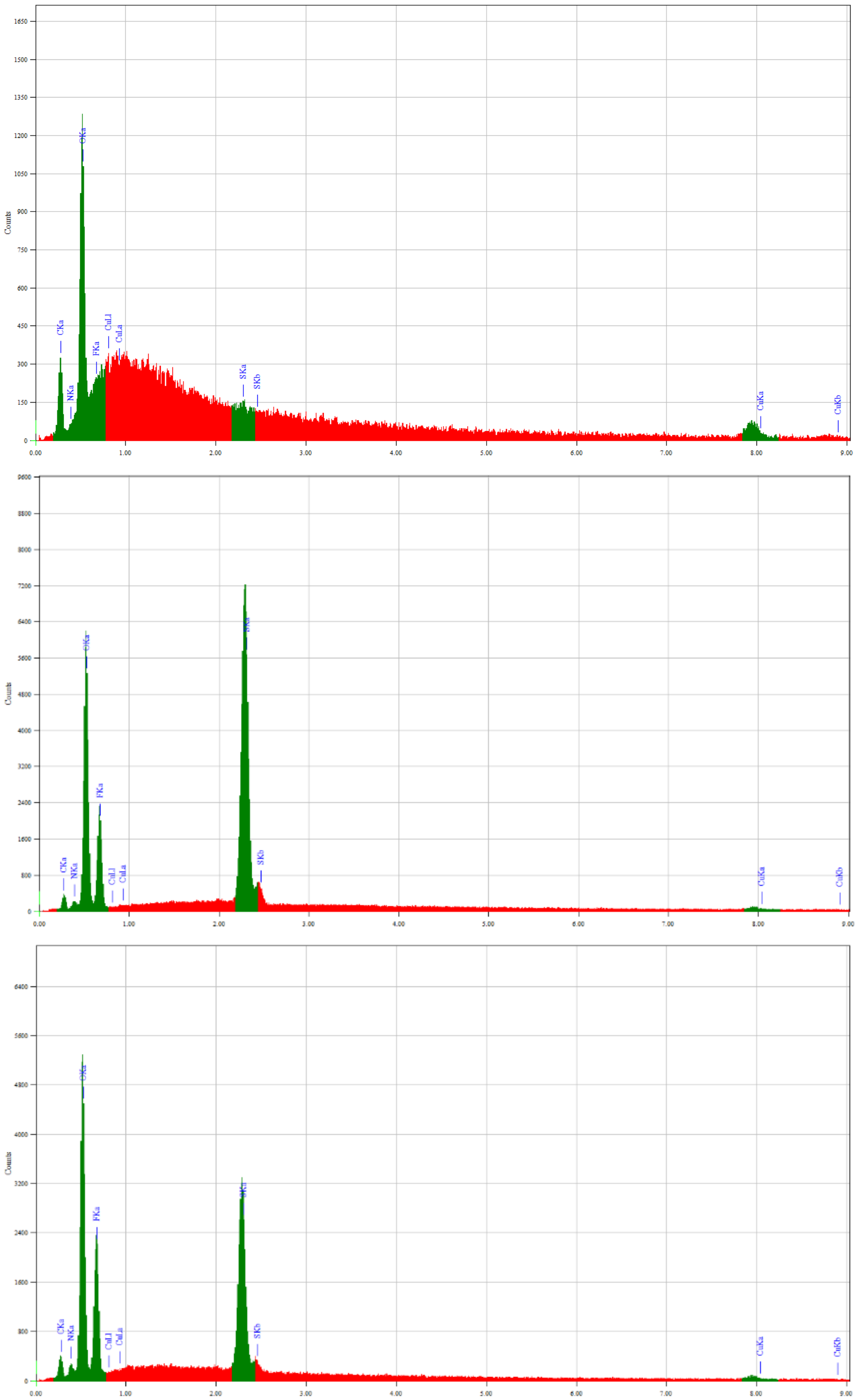

Figure S11. EDS spectra of pristine lithium anode (top) as well as post-cycled lithium anodes with PP (middle) and NATM@PP (bottom) separators.

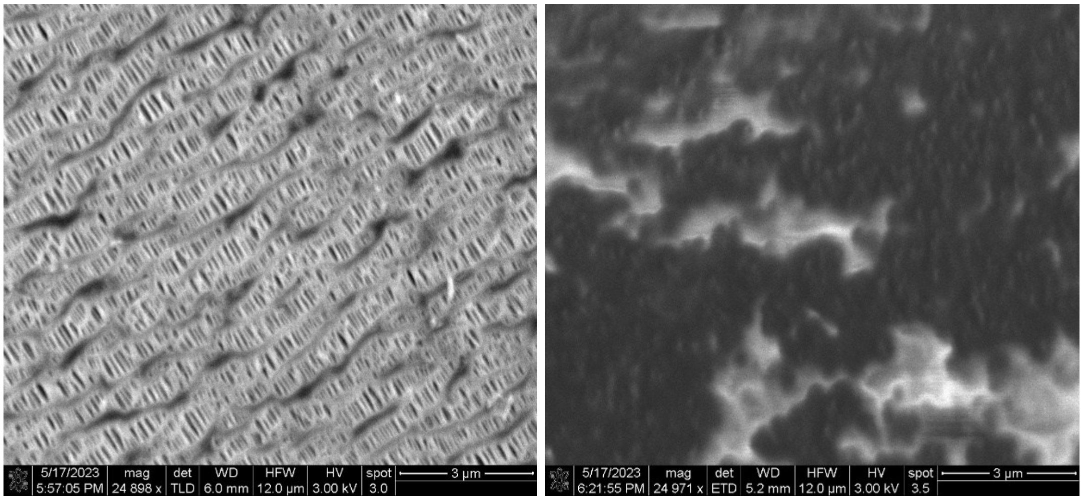

Figure S12. Additional SEM images of post-cycled PP (left) and NATM@PP (right) separators.

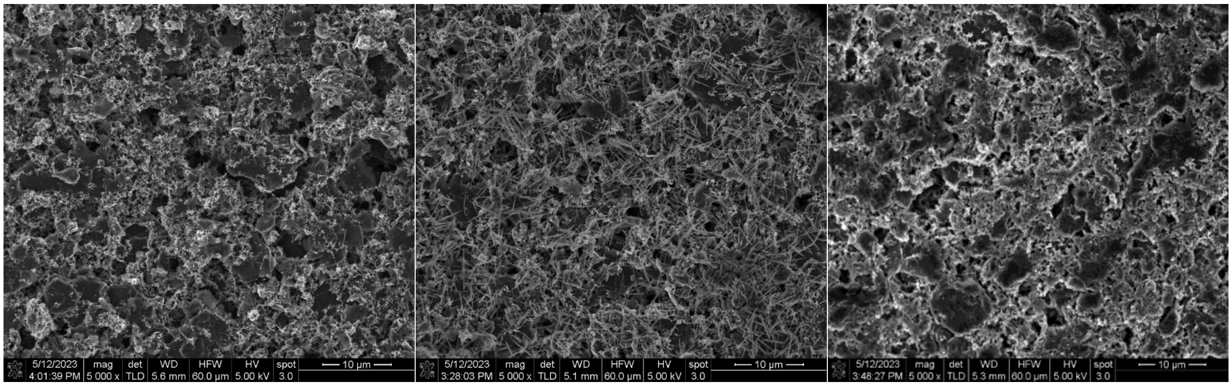

Figure S13. Additional SEM images of pristine (left) and post-cycled cathodes with PP (center) and NATM@PP (right) separators.

Table S1. Summary of all Z-fit parameters from Figure 3.

		R1 [Ω]	R2 [Ω]	Q2 [F.s^(a-1)]	a2	R3 [Ω]	Q3 [F.s^(a-1)]	a3	Q4 [F.s^(a-1)]	a4	s5 [Ω.s^(-1/2)]
Before	PP	3.65	26.04	2.36E-05	0.76	20.75	2.94E-03	0.859	0.1104	0.72	0.7689
	Gr@PP	3.58	26.71	2.82E-05	0.75	35.13	7.08E-03	0.804	0.0234	1	7.43
After	PP	10.2	10.24	1.24E-05	0.77	6.68	9.11E-03	0.627	0.1954	0.74	-1.4
	Gr@PP	14	4.252	1.30E-02	0.52	7.167	1.05E-04	0.613	0.1225	0.72	-2.914
